# Dual-color STED microscopy reveals a sandwich structure of Bassoon and Piccolo in active zones of adult and aged mice

**DOI:** 10.1038/srep27935

**Published:** 2016-06-20

**Authors:** Hiroshi Nishimune, Yomna Badawi, Shuuichi Mori, Kazuhiro Shigemoto

**Affiliations:** 1 Department of Anatomy and Cell Biology, University of Kansas School of Medicine, Kansas City, 66160, USA; 2 Department of Geriatric Medicine, Tokyo Metropolitan Institute of Gerontology, Itabashi-ku, Tokyo 173-0015, Japan

## Abstract

Presynaptic active zones play a pivotal role as synaptic vesicle release sites for synaptic transmission, but the molecular architecture of active zones in mammalian neuromuscular junctions (NMJs) at sub-diffraction limited resolution remains unknown. Bassoon and Piccolo are active zone specific cytosolic proteins essential for active zone assembly in NMJs, ribbon synapses, and brain synapses. These proteins are thought to colocalize and share some functions at active zones. Here, we report an unexpected finding of non-overlapping localization of these two proteins in mouse NMJs revealed using dual-color stimulated emission depletion (STED) super resolution microscopy. Piccolo puncta sandwiched Bassoon puncta and aligned in a Piccolo-Bassoon-Piccolo structure in adult NMJs. P/Q-type voltage-gated calcium channel (VGCC) puncta colocalized with Bassoon puncta. The P/Q-type VGCC and Bassoon protein levels decreased significantly in NMJs from aged mouse. In contrast, the Piccolo levels in NMJs from aged mice were comparable to levels in adult mice. This study revealed the molecular architecture of active zones in mouse NMJs at sub-diffraction limited resolution, and described the selective degeneration mechanism of active zone proteins in NMJs from aged mice. Interestingly, the localization pattern of active zone proteins described herein is similar to active zone structures described using electron microscope tomography.

Synaptic transmission is initiated by the arrival of action potentials to nerve terminals that open voltage-gated calcium channels (VGCCs) causing calcium influx and synaptic vesicle fusion to the presynaptic membrane. Proteins essential for synaptic transmission have been identified, however, the molecular architecture of this machinery has not been fully resolved. Presynaptic active zones are sites of synaptic vesicle accumulation and release, which was revealed using transmission electron microscopy^1^. Freeze fracture electron microscopy and electron microscope tomography have revealed the ultrastructure of active zones, but the molecular components of these structures remain unidentified^2^,^3^,^4^,^5^,^6^,^7^,^8^,^9^. Immunoelectron microscopy has advanced the analysis of molecular architecture of active zone proteins at presynaptic terminals^10^,^11^,^12^,^13^,^14^.

Super resolution microscopy techniques (for example, STED, Photoactivated Localization Microscopy (PALM) and stochastic optical reconstruction microscopy (STORM)) have improved the resolving power of light microscopy to under 50 nm and are being utilized to reveal the molecular architecture of presynaptic terminals^15^,^16^,^17^,^18^. These light microscopy techniques are more suitable for analyzing multiple proteins, relative locations of proteins, and a large number of synapses from multiple animals compared to electron microscopy methods. For example, these techniques have been used to resolve the three-dimensional distribution patterns of active zone proteins in *Drosophila* NMJs^19^,^20^,^21^,^22^,^23^. Furthermore, super resolution microscopy has been used to uncover the relative location of the active zone proteins, synaptic proteins, and pre- and post-synaptic proteins in central nervous system synapses of mice^24^,^25^,^26^,^27^. Mouse NMJs are ideal for analyzing active zones of mammalian synapses because NMJs are large, contain a few hundred active zones per synapse, and are flat and well suited for imaging based analysis^28^,^29^. However, active zone specific proteins in mammalian NMJs have not been analyzed using super resolution microscopy.

Using confocal microscopy, we previously described the distribution pattern of the active zone proteins Bassoon, Piccolo, and P/Q-type VGCCs in mouse NMJs as numerous small puncta distributing as discrete protein accumulations^28^,^29^,^30^. P/Q-type VGCCs are presynaptic calcium channels that are essential for synaptic transmission in adult NMJs^31^,^32^,^33^. Piccolo and Bassoon are large molecular weight active zone specific proteins that are essential in the maintenance of active zone structures^34^,^35^,^36^,^37^,^38^,^39^, and are involved in gathering synaptic vesicles, controlling synaptic transmission efficiency^39^,^40^,^41^,^42^,^43^, assembling presynaptic f-actin^44^, and controlling the function of VGCCs^30^,^41^. Importantly, we identified the molecular mechanism for organizing NMJ active zones, which consists of the interaction between the muscle-derived extracellular matrix molecule laminin β2, its specific receptor P/Q-type VGCC, and cytosolic active zone protein Bassoon^28^,^45^. The density of NMJ active zones within individual NMJs remains constant during postnatal development while NMJ size increases by three-fold^29^. However, the active zone density decreases in aged mice and aged rats compared to the active zone density in adult NMJs^29^,^30^. However, these analyses were limited by the diffraction-limited resolution of confocal microscopy. Here, we used STED microscopy to reveal the molecular architecture of active zones at sub-diffraction limited resolution. Bassoon and Piccolo are often analyzed together due to their protein structure similarity^43^,^46^ and are reported to colocalize in NMJs^29^ and CNS synapses^46^,^47^,^48^. However, we identified an unexpected non-overlapping side-by-side distribution pattern of these proteins in mouse NMJs.

## Results

### STED analysis of active zone proteins in mouse NMJs

**Figure 1:**
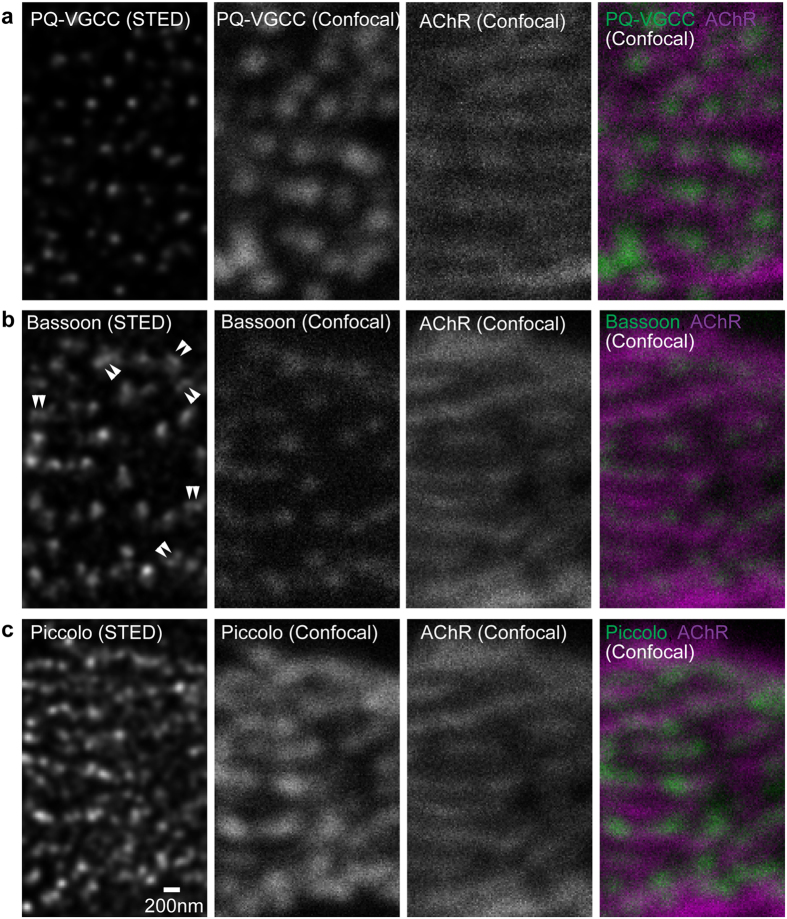
STED microscopy revealed distribution patterns of active zone proteins in an *en face* view of adult mouse NMJs. Fluorescent immunohistochemistry was used to visualize (**a**) P/Q-type voltage-gated calcium channels pore forming α subunit (PQ-VGCC), and active zone proteins (**b**) Bassoon and (**c**) Piccolo in NMJs of eight-month-old wild-type mice. STED microscopy images (far left column, deconvolved images) demonstrated significantly improved resolution of these three proteins compared to confocal microscopy images (second column from the left) in single optical plane images parallel to the presynaptic membrane. Double arrowheads in the Bassoon STED image indicate examples of doublet Bassoon puncta that were not resolved in confocal images. Confocal images revealed an alignment of these proteins with postsynaptic junctional folds, which was visualized as bright lines of α-bungarotoxin labeled acetylcholine receptors (most right column, magenta = AChR, green = active zone proteins). Scale bar: 200 nm.

**Figure 2:**
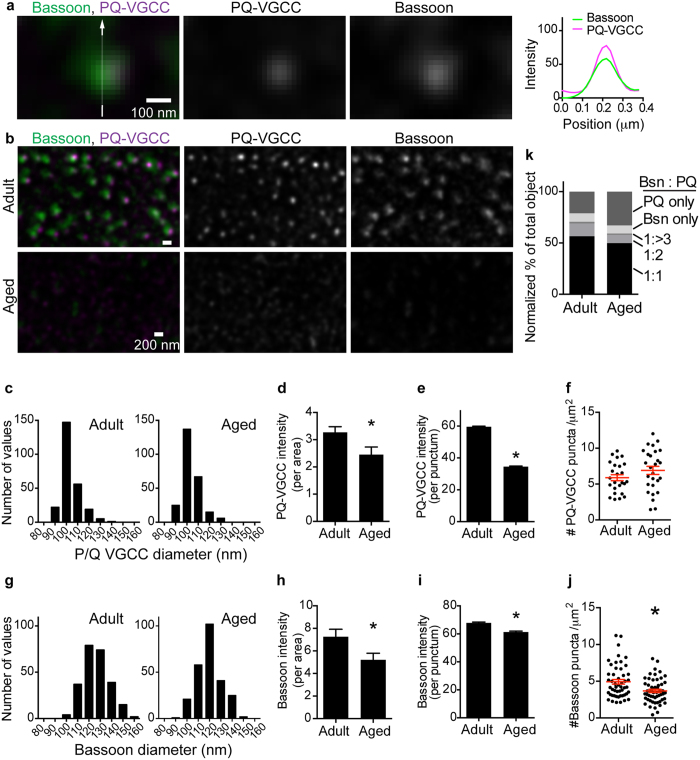
Bassoon colocalizes with P/Q-type VGCCs. (**a**) A representative dual-color STED micrograph of high magnification and single optical plane images parallel to the presynaptic membrane. A cluster of Bassoon (green) and P/Q-type VGCCs (magenta) overlapped near completely in NMJs of eight-month-old mice. The signal intensity profile is shown on the right. (**b**) The distribution pattern of Bassoon and P/Q-type VGCCs in NMJs of eight-month-old (adult) and 29-month-old (aged) mice visualized using STED microscopy at low magnification. Images in a and b are deconvolved images. Scale bars: 100 nm for a, and 200 nm for b. (**c**,**g**) Frequency distribution graphs of P/Q type-VGCC and Bassoon puncta size. The mean FWHM sizes of P/Q-type VGCC were 103.4 ± 8.5 nm for adult and 103.3 ± 8.3 nm for aged NMJs, and the FWHM of Bassoon was 126.4 ± 11.8 nm for adult and 123.0 ± 10.1 nm for aged NMJs (mean ± standard deviation). A total of 250 puncta in 25 NMJs from five mice at each age were quantified. (**d**,**h**) Signal intensity per area and (**e**,**i**) signal intensity per puncta decreased significantly for P/Q-type VGCC and Bassoon in NMJs from aged mice compared to NMJs from adult mice. (**f**,**j**) Puncta density decreased significantly in NMJs from aged mice for Bassoon but not for PQ-type VGCC compared to that in NMJs from adult mice. Each dot represents one NMJ, and red whiskers show the mean ± S.E.M. An asterisk indicates significance by unpaired t-test (p < 0.05). (**k**) Normalized ratio of calcium channel and Bassoon puncta colocalization pattern. Bassoon puncta overlapped with PQ-type VGCC puncta at a 1:1 ratio in the majority of cases and was present in fewer cases at a 1:2 ratio. The ratio of P/Q-type VGCC puncta without Bassoon increased in NMJs from aged mice. A total of 25 to 27 NMJs from five mice at eight or 29 months of age were measured. The Bassoon graph consisted of 51 to 54 NMJs by pooling data from PQ-VGCC-Bassoon images and Piccolo-Bassoon STED images. For (**e**,**i**,**k**) n = 2638 to 3350 puncta were analyzed.

Distribution patterns of active zone proteins in NMJs of adult wild-type mice (eight months old) were compared between time-gated STED microscopy^49^,^50^ and confocal microscopy. The micrographs are an *en face* view of NMJs and are parallel to the plane of the plasma membrane of presynaptic terminals. Adult NMJs rely primarily on presynaptic P/Q-type VGCCs for synaptic transmission^31^,^32^,^33^. In adult NMJs, P/Q-type VGCCs were distributed in a punctate pattern that aligned with bright lines of α-bungarotoxin staining pattern as detected via confocal microscopy (Fig. 1). These bright lines of α-bungarotoxin identifies postsynaptic junctional folds^51^. The alignment of VGCC puncta with junctional folds is consistent with the alignment of presynaptic active zones with junctional folds observed using electron microscopy^52^ and electron tomography^9^ because these calcium channels are thought to be in or near the active zones. STED micrographs revealed puncta size and distribution pattern at sub-diffraction limited resolution in single optical plane images. In STED images, the P/Q-type VGCC staining pattern in NMJs from adult mice consisted of smaller puncta with a mean full-width half maximum (FWHM) of 103.4 ± 8.5 nm compared to the same puncta in confocal images exhibiting a mean FWHM of 244.04 ± 25.5 nm (mean ± standard deviation, Figs 1 and 2).

The distribution pattern of the active zone specific protein Bassoon is characterized by small discrete puncta in nerve terminals that align with postsynaptic junctional folds in NMJs^28^,^29^. This distribution pattern was reconfirmed in the current study (Fig. 1). In STED images, Bassoon was present in puncta than were smaller than the puncta observed in confocal images. Bassoon puncta in NMJs from adult mice exhibited an average FWHM of 126.4 ± 11.8 nm in STED images compared to the same puncta in confocal images exhibited a FWHM of 241.5 ± 29.4 nm (mean ± standard deviation, Figs 1 and 2). Some Bassoon puncta were present as two-paired puncta in close proximity, which were not previously detected in confocal microscopy images.

**Figure 3:**
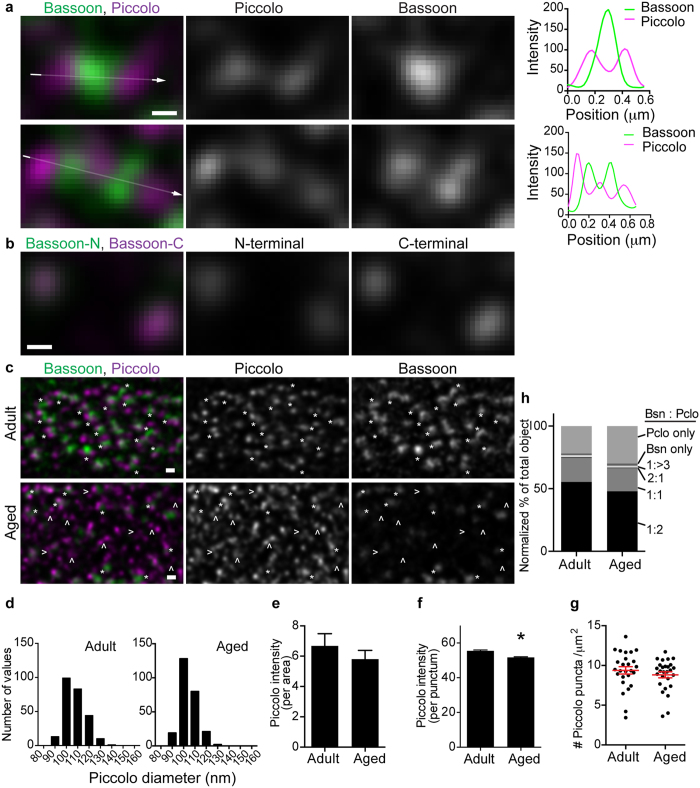
Piccolo and Bassoon aligned in a side-by-side pattern. (**a**) Representative dual-color STED micrographs show clusters of Piccolo (magenta) and Bassoon (green) in images parallel to the presynaptic membrane from NMJs of eight-month-old mice. Piccolo puncta were spaced in approximately 100 nm distance and sandwiched Bassoon puncta as a pair (top) or in repeats (bottom). The signal intensity profiles support this distribution pattern (far right). (**b**) A representative dual-color STED micrograph of Bassoon stained with different antibodies. One antibody binds to Bassoon near the N-terminus and the other near the C-terminus. These two signals overlapped near completely in images parallel to the presynaptic membrane. (**c**) A low magnification STED micrograph of Piccolo and Bassoon in NMJs of eight-month-old (adult) and 29-month-old (aged) mice. Many Piccolo puncta were present in the Piccolo-Bassoon-Piccolo alignment pattern in adult NMJs (asterisks), but the Bassoon signal was significantly reduced in aged NMJs (arrow heads). Images in (**a–c**) are deconvolved images. Scale bars: 100 nm for (**a**,**b**) 200 nm for (**c**). (**d**) Frequency distribution graphs of Piccolo puncta size. The mean FWHM was 107.6 ± 9.7 nm for adult and 104.0 ± 8.0 nm for aged NMJs (mean ± standard deviation). Two hundred fifty puncta in 25 NMJs from five mice at each age were quantified. Piccolo signal (**e**) intensity per area was similar, but (**f**) intensity per puncta decreased significantly in aged NMJs compared to adult NMJs. (**g**) Piccolo puncta density remained similar between adult and aged NMJs. Each dot represents one NMJ, and red whiskers indicate the mean ± S.E.M. (**h**) Normalized ratio of Piccolo and Bassoon puncta distribution pattern. In the majority of active zones, Piccolo and Bassoon puncta were present at a 2:1 ratio, but they were present at a 1:1 ratio in fewer active zones. In aged NMJs, the ratio increased for Piccolo puncta without Bassoon. Twenty-six to 27 NMJs from five mice at eight and 29 months of age were measured. For f and h: n = 2349 to 3126 puncta were analyzed.

The active zone specific protein Piccolo was present in a somewhat diffused and connected staining pattern compared to the distribution patterns of P/Q-type VGCCs and Bassoon in confocal microscopy images. Although expression was diffuse, Piccolo overlapped with post-synaptic junctional folds (Fig. 1). However, single optical plane STED images significantly improved the resolution of the Piccolo distribution pattern. Piccolo staining revealed discrete puncta residing very closely in STED images that were not previously detected in confocal microscopy images. Piccolo in NMJs of adult mice distributed in small puncta with an average FWHM of 107.6 ± 9.7 nm in STED images compared to the same puncta in confocal images exhibiting a FWHM of 249.5 ± 27.7 nm (mean ± standard deviation). The Piccolo puncta size was similar to the size of P/Q-type VGCC puncta in STED images (Figs 1 and 3). Interestingly, Piccolo puncta were more numerous than P/Q-type VGCC puncta and existed in pairs or groups in NMJs (Figs 1 and 3). These distribution patterns of P/Q-type VGCC, Bassoon, and Piccolo in adult NMJs suggest a discrete distribution pattern of active zones within one nerve terminal, which is consistent with active zones detected in mouse NMJs by electron microscopy^29^,^52^.

### Co-localization of Bassoon and VGCC

Next, we analyzed the colocalization of Bassoon and P/Q-type VGCC using dual-color STED microscopy images of the *en face* view of NMJs from adult mice. Fifty-six percent of Bassoon puncta colocalized with P/Q-type VGCC puncta at a 1:1 ratio (Fig. 2A,B,K). However, 14% of Bassoon puncta were found to colocalize in a combination of one Bassoon punctum with two or more P/Q-type VGCC puncta (Fig. 2K). These results are consistent with previous reports by us and others demonstrating both direct and indirect interactions between Bassoon and calcium channels^45^,^53^. Some Bassoon puncta (8.3%) were identified alone without P/Q-type VGCC. These results indicate that the majority of Bassoon puncta (70%) are localized near P/Q-type VGCCs, which is the site of Ca^2+^ influx.

### Piccolo-Bassoon-Piccolo distribution

Unexpectedly, Bassoon and Piccolo puncta did not overlap in dual-color STED images of the *en face* view of NMJs from adult mice, instead, the two proteins resided side-by-side in aligned puncta. This Piccolo distribution pattern was not resolved in previous confocal images. Piccolo puncta sandwiched a Bassoon punctum and formed a Piccolo-Bassoon-Piccolo structure in presynaptic terminals (Fig. 3A top). In some cases, this Piccolo-Bassoon-Piccolo structure was repeated in tandem (Fig. 3A bottom). Piccolo puncta were separated by approximately 100 nm, and Bassoon puncta filled the space between the two Piccolo puncta. In comparison, when Bassoon protein was labeled with two different antibodies, one recognizing close to the N-terminus and the other recognizing close to the C-terminus of this large 3942 amino acid protein (NCBI, NP_031593), the dual-color STED images overlapped in most areas of the *en face* view of presynaptic terminals (Fig. 3B). This result suggests that dual-color STED microscopy can be used to analyze the relative locations of two proteins without concern for chromatic aberration or the size of antibodies used for detection (~15 nm for each antibody). In other words, the non-overlapping side-by-side localization pattern of Bassoon and Piccolo is not the result of image drift induced by different wavelengths used for detection or the additional distance caused by antibody binding. Bassoon and Piccolo puncta rarely overlapped, and 55.4% of Bassoon puncta was found to group and align with Piccolo puncta in a 1:2 ratio (Fig. 3A,C,H). A smaller portion of Bassoon puncta (19.2%) were observed to group and align with Piccolo puncta in a 1:1 ratio, and only 22.0% of puncta contained only Piccolo (Fig. 3H). These data suggest that Bassoon and Piccolo form a structural unit in active zones characterized by a side-by-side distribution pattern.

### Reduction of active zone proteins in aged NMJs

**Figure 4:**
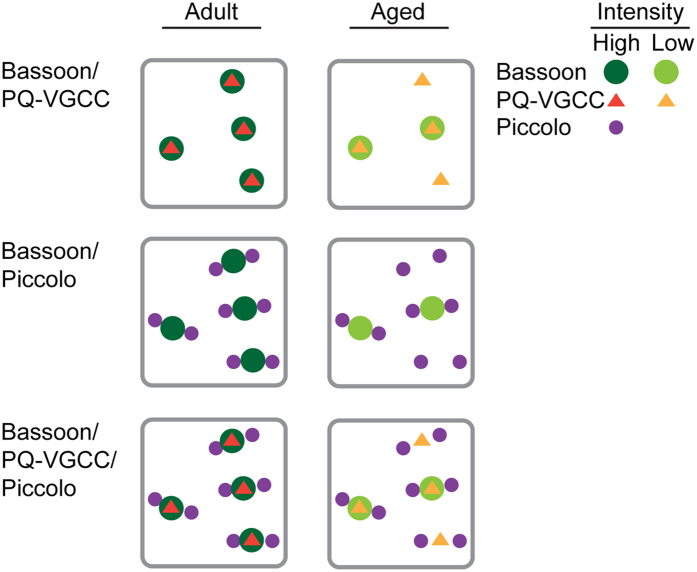
Summary of the current study and a theoretical combination figure. In NMJs from aged mice, Bassoon exhibited a decrease in puncta density and signal intensity, P/Q-type VGCC exhibited a decrease in puncta intensity, but Piccolo puncta density in NMJs from aged mice was similar to puncta density in NMJs from adult mice.

Are these active zone proteins maintained in NMJs of aged mice? The survival rate of C57 BL/6 mice significantly declines beyond 2 years of age^54^, thus, 29-month-old mice were analyzed as aged. NMJs from aged mice were analyzed using dual-color STED microscopy with the same conditions as aforementioned analyses of eight-month-old adult mice. In NMJs from aged mice, P/Q-type VGCCs exhibited a significant decrease in signal intensity per area (adult, 3.3 ± 0.2; aged, 2.5 ± 0.3 arbitrary units, unpaired t-test p = 0.0244, n = 25 (adult), 27 (aged) NMJs) and signal intensity per punctum (adult, 59.7 ± 0.4; aged, 36.3 ± 0.4 arbitrary units, unpaired t-test p < 0.0001, n = 2638 (adult), 3340 (aged) puncta) (Fig. 2B,D,E). However, the puncta size (FWHM adult, 103.4 ± 8.5 nm; aged, 103.3 ± 8.3 nm) (mean ± standard deviation) and the puncta density (adult, 5.9 ± 0.4; aged, 6.9 ± 0.6 puncta/μm^2^) were not significantly different between NMJs from adult and aged mice (Fig. 2C,F). These results indicate that VGCC protein level decreased in most of the active zones during aging, but reduced amount of protein remained in the active zones in aged NMJs (Fig. 4).

Bassoon exhibited a significant decrease in signal intensity per area in NMJs from aged mice (adult, 7.3 ± 0.7; aged, 5.2 ± 0.6 arbitrary units, unpaired t-test p = 0.0228, n = 51 (adult), 54 (aged) NMJs) similar to the pattern observed in P/Q-type VGCCs (Fig. 2H). Furthermore, Bassoon exhibited a small but significant decrease in the size of the remaining puncta (FWHM adult, 126.4 ± 11.8 nm; aged, 123.0 ± 10.1 nm (mean ± standard deviation), unpaired t-test, p = 0.0005, n = 250 (adult), 250 (aged) puncta) and in the signal intensity per puncta (adult, 67.8 ± 0.6; aged, 61.3 ± 0.6 arbitrary units, unpaired t-test p < 0.0001, n = 3032 (adult), 3350 (aged) puncta) compared to NMJs from adult mice (Fig. 2G,I). Importantly, in contrast to VGCC, the density of Bassoon puncta decreased significantly in NMJs from aged mice (adult, 4.9 ± 0.3; aged, 3.7 ± 0.2 puncta/μm^2^, unpaired t-test p < 0.0019, n = 51 (adult), 54 (aged) NMJs) (Fig. 2J). These results suggest that the Bassoon protein was lost from many active zones during aging but remained present in a few active zones at slightly reduced levels compared to active zones in NMJs from adult mice (Fig. 4). This decrease in active zone proteins is not due to denervation of NMJs because the detection of the neuronal protein Bassoon at a reduced level indicates that nerve terminals were present in the analyzed NMJs from aged mice. The decrease in Bassoon protein in NMJs from aged mice is consistent with our previous studies^29^,^30^. Consistent with these alterations in P/Q-type VGCC and Bassoon protein level, the ratio of P/Q-type VGCC puncta without Bassoon increased in NMJs from aged mice (Fig. 2K).

In contrast to P/Q-type VGCCs and Bassoon, Piccolo did not demonstrate a significant decrease in signal intensity per area between NMJs from adult and aged mice (adult, 6.7 ± 0.8; aged, 5.8 ± 0.6 arbitrary units, unpaired t test p = 0.3793, n = 26 (adult), 27 (aged) NMJs) (Fig. 3E). However, similar to Bassoon, a small decrease in the size of Piccolo puncta was observed between NMJs from adult and aged mice (FWHM adult, 107.6 ± 9.7 nm; aged, 104.0 ± 8.0 nm (mean ± standard deviation), unpaired t-test p < 0.0001, n = 250 (adult), 250 (aged) puncta) and in the signal intensity per puncta (adult, 55.5 ± 0.5; aged, 51.7 ± 0.4 arbitrary units, unpaired t-test p < 0.0001, n = 2349 (adult), 3126 (aged) puncta) (Fig. 3D,F). Similar to P/Q-type VGCCs but in contrast to Bassoon, Piccolo did not demonstrate significant differences in puncta density in NMJs from aged mice (adult, 9.4 ± 0.5; aged, 8.8 ± 0.4 puncta/μm^2^, unpaired t test p = 0.3544, n = 26 (adult), 27 (aged) NMJs) (Fig. 3G). These results indicate that Piccolo protein level in NMJs from aged mice decreased slightly but remained comparable to the level in NMJs from adult mice. However, due to a decrease in Bassoon puncta density, some active zones were missing Bassoon puncta in between the Piccolo puncta pairs (Fig. 3C). As a consequence, the ratio of solitary Piccolo puncta increased in NMJs from aged mice, and the ratio of Piccolo-Bassoon-Piccolo puncta in combination decreased (Fig. 3H). The analyzed NMJs from aged mice were not denervated because the detection of neuronal protein Piccolo indicated the presence of nerve terminals in these NMJs. In summary, these results identified that active zone proteins selectively decrease, and not by uniform degeneration, prior to NMJ denervation during aging (Fig. 4).

## Discussion

To our knowledge, this is the first study to reveal the distribution pattern of P/Q-type calcium channels in mammalian NMJs at sub-diffraction limited resolution and to describe the age-related alterations in NMJ active zone organization. P/Q-type VGCCs localized to puncta of 103 nm FWHM size in a discrete fashion throughout the motor nerve terminal and colocalized with the active zone specific protein Bassoon. Bassoon and Piccolo are active zone specific cytosolic proteins essential for active zone assembly in NMJs, ribbon synapses, and brain synapses^55^. These proteins have been proposed to colocalize and share some functions in active zones^43^,^46^. Unexpectedly, we identified in this study that Piccolo does not colocalize with Bassoon but is distributed in small discrete puncta of 107 nm FWHM size throughout motor nerve terminals. Importantly, Piccolo puncta sandwiched a Bassoon punctum in a side-by-side pattern, indicative of a functional unit in the active zone. These distribution patterns of active zone proteins at sub-diffraction limited resolution were not previously known using confocal microscopy analyses of mouse NMJs. Bassoon puncta size was quantified as 126 nm FWHM and was larger than P/Q-type VGCCs and Piccolo puncta.

The first analysis of active zone proteins using super resolution microscopy revealed the distribution pattern of Bruchpilot (nc82) in *Drosophila* NMJs using STED microscopy^19^. This initial analysis revealed a ring like image of Bruchpilot protein distribution pattern in each active zone. Further analysis using STED microscopy revealed a bouquet like distribution pattern of Bruchpilot, the location of Bruchpilot relative to calcium channels (Calcium channel α1 subunit Cacophony)^20^ and Rab3-interacting molecule-binding proteins (DRBPs)^21^, which are essential for neurotransmitter release in *Drosophila* NMJs. In addition, STORM analyses have resolved the three-dimensional distribution patterns of active zone proteins^19^,^20^,^21^,^22^,^23^ and quantified Bruchpilot protein quantity in *Drosophila* NMJs at sub-diffraction limited resolution^22^. Super resolution microscopy has resolved the relative location of the active zone proteins, synaptic proteins, and pre- and post-synaptic proteins in central nervous system synapses in mice^24^,^25^,^26^,^27^. In the current study, STED analysis of mouse NMJs revealed punctate and discrete distribution patterns (Bassoon, P/Q-type VGCCs) and a sandwich structure of active zone proteins (Piccolo-Bassoon-Piccolo). These results suggest that active zones in mouse NMJs consist of P/Q-type VGCCs, colocalizing Bassoon, and two separate puncta of Piccolo. However, Bassoon was not present in a bouquet like pattern in mouse NMJs like Bruchpilot at *Drosophila* NMJs. The similarities between mouse and *Drosophila* active zones include the punctate distribution pattern of VGCCs, and the colocalization of active zone specific proteins (Bassoon and Bruchpilot) with VGCC puncta. This study offers the opportunity to compare the distribution pattern of active zone specific proteins within synapses of central and peripheral nervous systems at the sub-diffraction limited resolution. Bassoon distribution pattern in mouse NMJs revealed using STED microscopy was similar to that of GABAergic inhibitory interneuron synapses in the mouse hippocampus revealed by 3D-STORM microscopy (Fig. 5 in ref. 56). Both synapses showed discrete punctate distribution patterns of Bassoon within one presynaptic terminal. Excitatory synapses of the mouse main olfactory bulb were analyzed using three-color 3D-STORM microscopy^24^. The synapses in the main olfactory bulb were small and included synapses about half the size of synapses in the cortex^24^. In these small synapses, Piccolo and Bassoon distributed in discrete punctate patterns similar to the pattern observed in NMJs (Fig. 4A,D in ref. 24). These two proteins appeared not to overlap completely (Fig. 4D in ref. 24), which suggested a similarity to the alternate distribution pattern of Piccolo and Bassoon in NMJs.

Bassoon and Piccolo are closely related multi-domain proteins that share two zinc finger domains and three coiled-coil domains^55^. Therefore, the two proteins are considered to be functionally related. Indeed, the two proteins arrive at nascent synapses together as a Piccolo-Bassoon transport vesicle^57^,^58^, and they both bind to E3 ubiquitin ligase Siah1 and negatively regulate Siah1 in the regulation of synapse integrity^59^. However, differences exist between Bassoon and Piccolo. Piccolo has a PDZ domain and two C2-domains, which do not exist in Bassoon^55^. Bassoon and Piccolo both bind to common proteins including ELKS and Siah1^55^, but Piccolo binds to many other proteins including actin, L-type VGCC, and Synapsin I^44^,^60^,^61^. Piccolo negatively regulates synaptic vesicle exocytosis through presynaptic F-actin assembly^44^ and modulation of Synapsin 1a^61^. Interestingly, these functions are not shared by Bassoon, which demonstrates a unique role for Piccolo in active zones^61^. Meanwhile, Bassoon binds to P/Q-type VGCCs directly or indirectly^30^,^53^ and modulates ion channel function^30^, while Piccolo has not been reported to modulate P/Q-type VGCC function. Furthermore, Bassoon and Piccolo distribute differently in some active zones of Calyx of Held synapses^46^. Only 60% of Bassoon and Piccolo puncta overlap. The remaining Bassoon and Piccolo puncta do not colocalize, suggesting that these two proteins are distributed differently to produce heterogeneous functional properties in different active zones within one synapse. Together, these unique roles for Bassoon or Piccolo, different interacting proteins, and the partial non-overlapping distribution pattern in Calyx of Held active zones are consistent with the non-overlapping distribution pattern of Bassoon and Piccolo identified in NMJs in the current study. The sandwich-like distribution pattern of Bassoon and Piccolo may relate to the interaction with different VGCCs^30^,^53^,^60^ and the different locations of presynaptic VGCCs proposed as a slot hypothesis by Tsien’s group^62^. The separate distribution patterns of Bassoon and Piccolo in NMJ active zones may be beneficial for exerting different functions, including the modulation of VGCC function by Bassoon and the regulation of synaptic vesicle exocytosis by Piccolo.

Freeze-fracture electron microscopy revealed a unit structure in active zones. The NMJs exhibit parallel rows of large intramembranous particles on the P-face (the interior of the cytosolic half of a plasma membrane) of presynaptic membranes in many species including humans and mice^2^,^3^,^4^,^5^. These findings suggested that an active zone is composed of a parallel array of 10–12 nm intramembranous particles arranged in four rows, with each active zone containing 20 of these intramembranous particles^2^. This proposed structure matches well with the active zone structure in mouse NMJs identified by electron microscope tomography analysis^9^. Macromolecules of active zone components connect to each other in structures coined “beams”, “ribs”, and “pegs” to the transmembrane channel structure (similar to the intramembranous particles detected by freeze-fracture electron microscopy) and to the docked synaptic vesicles in the active zones in nerve terminals (Supplementary Fig. 4a). A more detailed three-dimensional structure of active zones was revealed in frog NMJs using electron microscope tomography^6^,^7^,^8^, but it was not compared to the STED images in this study due to the structural differences in active zones between frog and mouse NMJs^9^.

It is tempting to speculate on the similarities between the STED microscopy results and the electron microscope tomography results. We detected a sandwich distribution pattern of Piccolo-Bassoon-Piccolo proteins that appeared to form a unit with P/Q-type VGCCs beneath Bassoon. Nagwaney *et al*. reported a model structure of beams-ribs-beams with channel proteins beneath the ribs as a unit of active zones^9^. Interestingly, this beams-ribs-beams structural unit matches with the Piccolo-Bassoon-Piccolo structure described in this study. Two Piccolo puncta were separated by ~100 nm according to STED images (Fig. 3), which is similar to the distance between two ribs in Nagwaney’s model (~83 nm) (Supplementary Fig. 4). Furthermore, the channel structures beneath the ribs matched with P/Q-type VGCC puncta colocalizing with Bassoon puncta. These similarities support the hypothesis that Piccolo-Bassoon-Piccolo with P/Q-type VGCC is a functional unit in the active zones in mouse NMJs.

In NMJs of aged mice, P/Q-type VGCC and Bassoon protein levels decreased significantly compared to adult NMJs. This result suggested that one potential cause of functional decline in aged NMJs is a significant loss of P/Q-type VGCC proteins in most active zones and a complete loss of Bassoon in many of the active zones. The aged active zones exhibited a reduced level of P/Q-type VGCC protein and lacked accompanying Bassoon proteins. The lack of Bassoon enhances the inactivation of P/Q-type VGCC based on our previous study^30^. These findings imply that the active zones of aged mouse NMJs function less efficiently due to two reasons: reduced protein level and reduced function of P/Q-type VGCCs. Interestingly, the active zone specific proteins, Bassoon and Piccolo, were differently decreased in NMJs of aged mice, indicating a selective process of active zone degeneration during aging. This is the first report of a selective degeneration of NMJ active zone proteins during aging, and further investigation is needed to reveal the aging related loss in neuromuscular function. The novel distribution patterns of active zone proteins revealed in this study will aid in such investigations.

## Methods

### Animals

Animal experiments were carried out in accordance with the animal care and use protocol approved by the Institutional Animal Care and Use Committee of Tokyo Metropolitan Institute of Gerontology (TMIG) and in accordance with the Guidelines for the Care and Use of Laboratory Animals of TMIG. C57BL/6NCr wild-type mice were purchased from CLEA Japan, Inc. (Tokyo, Japan) and maintained in the animal facility at TMIG until analysis. Five female mice were analyzed at adult (eight months old) and aged (29 months old) stages.

### Antibodies

#### Bassoon

(Mouse monoclonal antibody, SAP7F407, Enzo Lifesciences, Farmingdale, NY, USA, antibody registry antibody ID#: AB_1659574, used at a 1:600 dilution) This commercially available antibody binds to Bassoon near to the N-terminus and detects a punctate pattern in cultured rat hippocampal neurons at synapses labeled by an anti-synaptophysin antibody^35^. We and others have reported that this antibody binds to mouse NMJs^28^,^29^,^45^,^63^, and our previous study demonstrated that the Bassoon signal was absent in denervated NMJs^29^. The reduced level of Bassoon signal in aged NMJs was detected using this monoclonal antibody, thus there is a possibility that the reduction of signal intensity may result from a change in the post-translational modification of Bassoon.

#### Bassoon

(Rabbit polyclonal antibody, Synaptic systems, Goettingen, GERMANY, antibody registry antibody ID#: AB_887698 and AB_2274984, used at a 1:1000 dilution) This commercially available antibody specifically binds to Bassoon near the C-terminus and detects a punctate pattern in hippocampal neurons at synapses labeled by an anti-synaptophysin antibody (Manufacturer’s information).

#### Piccolo

(Rabbit polyclonal antibody, Synaptic systems, Goettingen, GERMANY, antibody registry antibody ID#: AB_887759, used at a 1:2000 dilution) The commercially available Piccolo antibody detects ~520 and ~65 kDa proteins in Western blots of mouse, rat, and chicken synaptic junctional proteins^64^. By immunocytochemistry, this antibody detects a punctate pattern in cultured rat hippocampal neurons that colocalize with the active zone specific protein Bassoon^65^. By immunohistochemistry, we and others have reported that this antibody stains rat NMJs and embryonic mouse NMJs^29^,^45^,^66^.

#### P/Q-type voltage-gated calcium channel

(Rabbit poly clonal antibody, Synaptic systems, Goettingen, GERMANY, catalogue number 152203, used at a 1:1000 dilution). This commercially available antibody is raised against recombinant protein of rat Ca^2+^ channel α-1A subunit (1921–2212 amino acid) and affinity purified with the immunogen. The antibody is specific for Ca^2+^ channel α-1A (Cav2.1), and the specificity has been verified using knockout mice tissue (Supplementary Fig. 5 and manufacturer’s observation). Our previous study demonstrated that this antibody stains mouse NMJs by immunohistochemistry^30^.

Alexa Fluor 488-conjugated anti-mouse IgG2a and Alexa Fluor 555-conjugated anti-rabbit IgG (H + L) F(ab’)2 fragment secondary antibodies (used at a 1:1000 dilution), biotin conjugated α-bungarotoxin (used at a 1:3000 dilution), and Pacific Orange conjugated Streptavidin (used at a 1:1000 dilution) were obtained from Molecular Probes, Thermo Fisher Scientific (Carlsbad, CA, USA).

### Immunohistochemical analysis

Immunohistochemistry methods have been described previously^28^,^29^,^45^,^67^. In brief, mice were fixed by transcardiac perfusion with 2% paraformaldehyde in PBS. Sternocleidomastoid muscle was dissected and post-fixed in the same fixative at room temperature, cryoprotected in 20% sucrose/PBS at 4˚C overnight, frozen in Optimal Cutting Temperature compound (Sakura), and sectioned using a cryostat. Longitudinal sections were cut at 20 μm thickness and blocked in PBS containing 2% BSA, 2% normal goat serum, and 0.1% Triton X-100. Sections were incubated with primary antibodies and biotin conjugated α-bungarotoxin overnight at room temperature, washed with PBS, and incubated with a mixture of secondary antibodies and Pacific Orange conjugated Streptavidin for 2 hours at room temperature. After washes with PBS, the sections were mounted in Prolong Gold mounting medium (Life Technologies, Carlsbad, CA, USA) with No. 1.5 cover glass (Fisher Scientific, Pittsburgh, PA USA). No staining was observed when primary antibodies were omitted.

### STED microscopy

Images were obtained using a Leica TCS SP8 STED 3X with a 100× objective lens (HC PL APO 100×/1.40 Oil STED White) and immersion oil (Leica Type F, refractive index 1.5180). Dual-color STED images were obtained by sequentially scanning a single-optical-plane of a given area. Images were acquired with a pixel size of 20 nm and an image size of 19.4 μm × 19.4 μm. The pinhole was set at one airy unit. An STED laser (660 nm) was applied at 80% of maximum power with 3X distribution for Bassoon imaging. The Bassoon signal intensity was low, and using the STED 3X mode improved the signal to noise ratio of the obtained images. Emission lights were collected using the Leica acousto-optical beam splitter, tunable spectral detector, and the time-gated detection.

Alexa Fluor 488 dye was excited with a white light laser using a 488 nm wavelength and was depleted using the 660 nm STED laser. Alexa Fluor 555 dye was excited with a white light laser using a 561 nm wavelength and was depleted using the 660 nm STED laser. These two fluorescent dyes were selected for dual-color STED microscopy for the following reasons: (1) Alexa Fluor 488 and Alexa Fluor 555 dyes are stable without shifts in excitation wavelengths and reliably demonstrate the STED effect in our sample preparation conditions; (2) Alexa Fluor 568 and Alexa Fluor 514 dyes have demonstrated shifts in their excitation wavelengths in some sample preparation conditions and have been excited with the 660 nm STED laser (Leica technical information). All STED images were deconvolved using Huygens software (Scientific Volume Imaging B.V., Hilversum, the Netherlands). Huygens software uses a signal reassignment algorithm for deconvolution (not subtraction based de-blurring); therefore, deconvolved images are suitable for intensity measurements and comparisons between specimens stained and imaged under the same condition. (Manufacturer information from Vincent Schoonderwoert, PhD, Scientific Volume Imaging B.V.). The same deconvolution condition was applied to all images using the manufacturer’s recommended parameters for intensity measurements, including the Classic Maximum Likelihood Estimation (CMLE) algorithm, fixed values for maximum iteration number with low quality threshold value, signal to noise ratio, and background estimation radius. The images in the figures are deconvolved images without any further level adjustments or modifications. STED images without deconvolution and quantifications using images without deconvolution are shown in Supplementary Figs 1–3. Quantification results obtained from the raw STED images and the deconvolved STED images were very similar.

Immediately after acquiring the STED images, the same area was scanned using the confocal microscopy mode with a 405 nm UV laser to visualize the Pacific-Orange labeled α-bungarotoxin. This dye is not excited by the dual-color STED microscopy with the emission wavelengths described above or the 660 nm STED laser. The STED images and confocal images do not align at a sub-diffraction limited resolution (specification of the STED microscope given by the manufacturer). Thus, the α-bungarotoxin images obtained using the confocal microscopy mode were used only as a guideline to identify the location of NMJs in the STED images.

### Image analysis

A FWHM of each punctum was measured using the twin slicer mode of the Huygens software to determine the punctum size. A line crossing a punctum was drawn which included the background level on both sides in a grayscale STED image. In the intensity plot graph of the Huygens software, a vertical line was drawn from the maximum intensity point to the background level to measure the height of the intensity plot. At the half maximum height, a horizontal line was drawn to measure the FWHM of the punctum. Two hundred and fifty representative puncta were measured by analyzing 10 puncta in each NMJ, five NMJs per animal, and five animals in each group.

For intensity and density analyses, immunohistochemistry images were quantified using protocols similar to our previous analysis of NMJ active zones^29^. First, we restricted the analysis area to the presynaptic area overlying postsynaptic area visualized by α-bungarotoxin-labeled AChR clusters. Puncta intensity was quantified using MetaMorph image analysis software (Molecular Devices, Sunnyvale, CA). We used the MetaMorph function “auto threshold for light objects” to threshold the images as objectively as possible for image quantification. Briefly, this algorithm utilizes the distribution of a histogram of pixel gray values and defines a background as the gray values with the largest peak. The algorithm defines the objects as peaks of the histogram on the bright side of the background. The algorithm then locates a trough in the histogram between the objects and the background and defines a threshold value^68^. This automated method produced threshold values accurately covering puncta in STED images. Signal intensities of active zone proteins were quantified as intensity per area (defined as integrated signal intensity divided by area of α-bungarotoxin-labeled AChR cluster) or intensity per puncta (defined as the average signal intensity within one punctum). Puncta density was calculated by dividing the puncta number by area of the α-bungarotoxin-labeled AChR cluster. The puncta number was the number of thresholded objects inside the area of the α-bungarotoxin-labeled AChR cluster.

For puncta colocalization and distribution pattern analyses, STED images were overlaid as an RGB image and evaluated using the MetaMorph cell counter function to label and log quantified objects (puncta). Puncta colocalization was analyzed manually for the following reasons: (1) we aimed to reveal the number of puncta associated with Bassoon puncta; (2) some puncta did not overlap completely but were present in a side-by-side pattern; and (3) puncta sizes were different among Bassoon, Piccolo, and P/Q-type VGCC. For these reasons, colocalization analyses using co-efficient values were not suitable for this study. For dual-color STED images of Bassoon and P/Q-type VGCC, each Bassoon punctum was scored into one of the following five categories to quantify the number of P/Q-type VGCC puncta associated with the Bassoon puncta: one VGCC for one Bassoon, two VGCC for one Bassoon, three or more VGCC for one Bassoon, Bassoon only, and VGCC only. For dual-color STED images of Bassoon and Piccolo, each Bassoon punctum was binned into six categories to quantify the number of Piccolo puncta associated with the Bassoon puncta: one Piccolo for one Bassoon, two Piccolo for one Bassoon, three or more Piccolo for one Bassoon, one Piccolo for two Bassoon, Bassoon only, and Piccolo only. The same analytical methods were applied to images acquired from the adult and aged NMJs.

### Statistics

All statistics were performed using GraphPad Prism software version 6. Significance was assessed by unpaired t-test with two-tailed P value. The P and N values are described in the text. FWHM are shown as mean ± standard deviation, all other data are shown as the mean ± S.E.M.

## Additional Information

**How to cite this article**: Nishimune, H. *et al*. Dual-color STED microscopy reveals a sandwich structure of Bassoon and Piccolo in active zones of adult and aged mice. *Sci. Rep.* **6**, 27935; doi: 10.1038/srep27935 (2016).

## Supplementary Material

Supplementary Information
