# Supplementary material for: Dual-color STED microscopy reveals a sandwich structure of Bassoon and Piccolo in active zones of adult and aged mice
Source: Sci Rep. 2016 Jun 20;6:27935. doi: 10.1038/srep27935 (PMC4913281; doi:10.1038/srep27935)
Supplement: Supplementary Information [file srep27935-s1.pdf]

Supplementary Figures 1 - 5

Title: Dual-color STED microscopy reveals a sandwich structure of Bassoon and Piccolo in active zones of adult and aged mice.

Authors: Hiroshi Nishimune, Yomna Badawi, Shuuichi Mori, and Kazuhiro Shigemoto

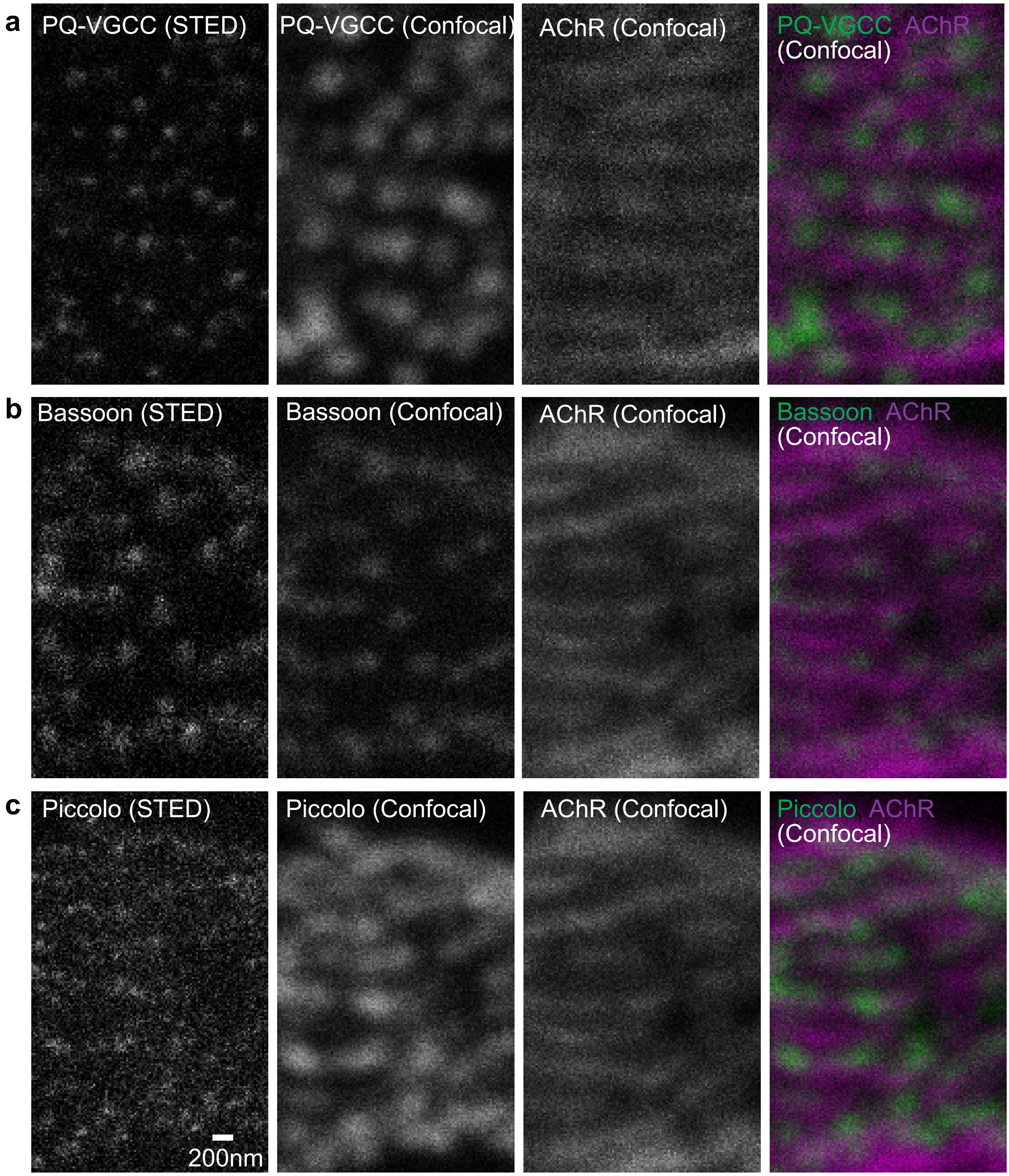

Supplementary Figure 1. STED microscopy images of figure 1 are shown without deconvolution. STED images (far left column) and confocal images (three columns from the right) in single optical plane images parallel to the presynaptic membrane are shown for (a) PQ-VGCC, and active zone proteins (b) Bassoon and (c) Piccolo in NMJs of eight-month-old wild-type mice. Scale bar: 200 nm.

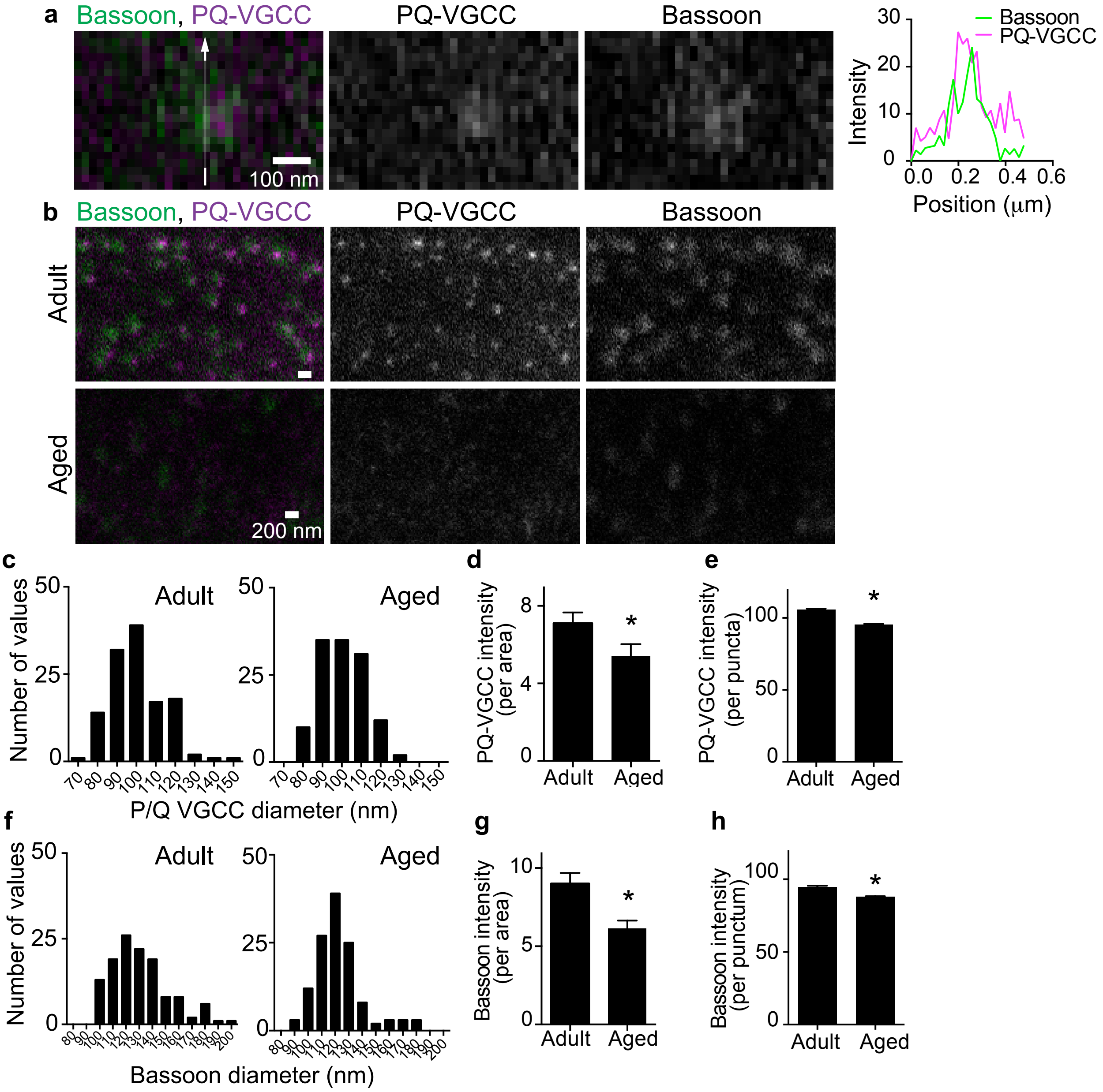

Supplementary Figure 2. (a, b) Images of figure 2 are shown without deconvolution. (c, f) FWHM, (d, g) signal intensity per area, and (e, h) signal intensity per puncta were quantified using the STED images without deconvolution. (a) A representative dual-color STED micrograph of high magnification and single optical plane images parallel to the presynaptic membrane. The signal intensity profile of the images supports the colocalization of the two proteins (far right). (b) The distribution pattern of Bassoon and P/Q-type VGCCs in NMJs of eight-month-old (Adult) and 29-month-old (Aged) mice visualized using STED microscopy at low magnification. Scale bars: 100 nm for a, and 200 nm for b. (c, f) Frequency distribution graphs of P/Q type-VGCC and Bassoon puncta size. The mean FWHM sizes of P/Q-type VGCC did not change (adult, 100.6 ± 13.5 nm; 100.5 ± 11.5 nm (aged), mean ± standard deviation, unpaired t-test, $p = 0.901$, $n = 125$ (adult), 125 (aged) puncta in 25 NMJs from five mice each). The mean FWHM of Bassoon decreased significantly (adult, 130.5 ± 22.2 nm; aged, 122.5 ± 18.0 nm, mean ± standard deviation, unpaired t-test, $p = 0.0019$, $n = 125$ (adult), 125 (aged) puncta in 25 NMJs from five mice each). P/Q-type VGCC exhibited significant decreases for (d) signal intensity per area (adult, 7.1 ± 0.5; aged, 5.4 ± 0.6 arbitrary units unpaired t test, $p = 0.0448$, $n = 25$ (adult), 27 (aged) NMJs from five mice each) and (e) signal intensity per puncta (adult, 105.9 ± 0.5; aged, 95.3 ± 0.6 arbitrary units, unpaired t test, $p < 0.0001$, $n = 2827$ (adult), 3516 (aged) puncta from five mice each) in NMJs from aged mice compared to NMJs from adult mice. Bassoon exhibited significant decreases for (g) signal intensity per area (adult, 9.0 ± 0.7; aged, 6.1 ± 0.5 arbitrary units, unpaired t test, $p = 0.0007$, 51 (adult), 54 (aged) NMJs from five mice each, data were pooled from PQ-VGCC-Bassoon images and Piccolo-Bassoon images) and (h) signal intensity per puncta (adult, 94.8 ± 0.7; aged, 87.8 ± 0.5 arbitrary units, unpaired t test, $p < 0.0001$, $n = 3839$ (adult), 3938 (aged) puncta from five mice each) in NMJs from aged mice compared to NMJs from adult mice.

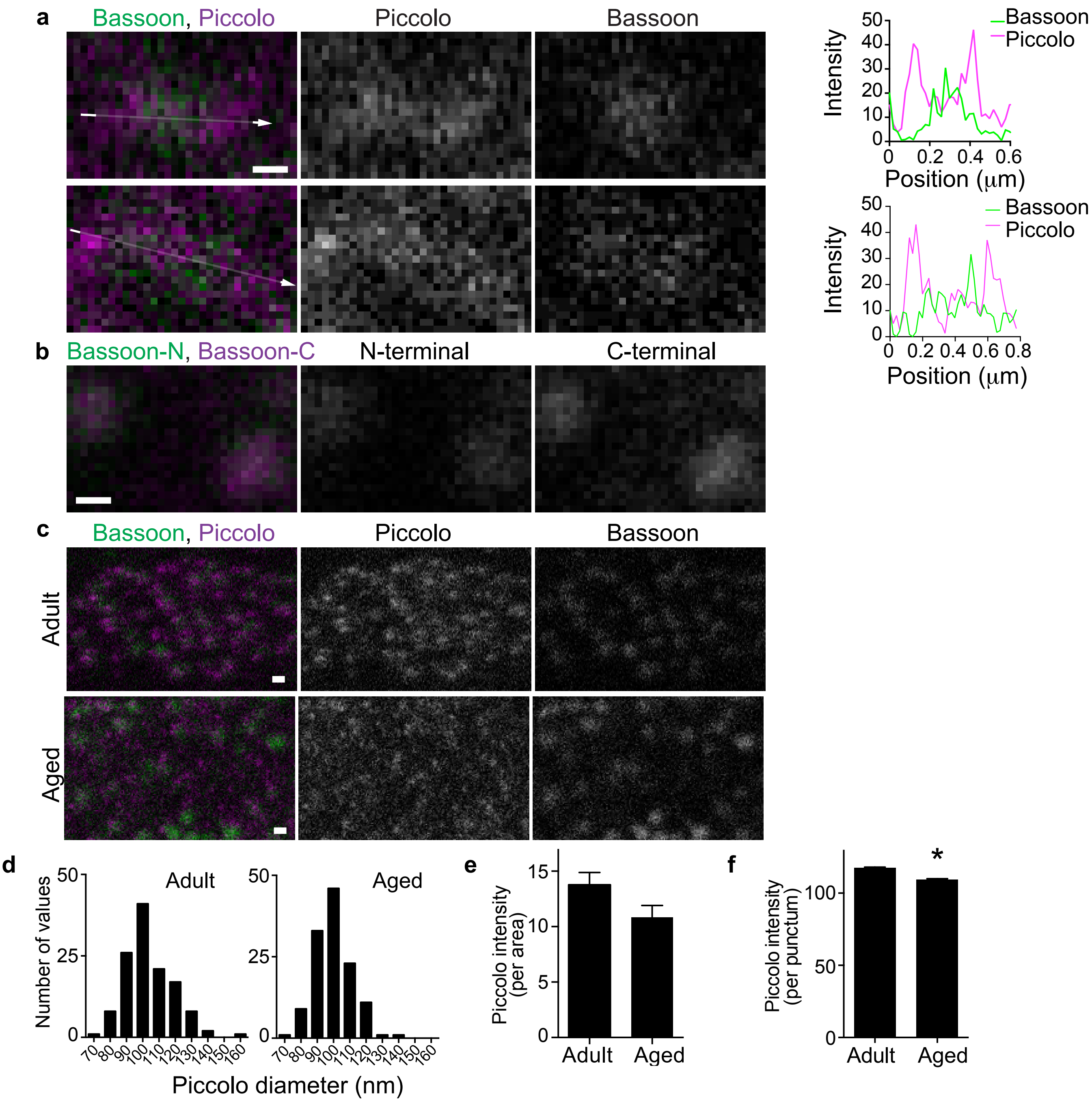

Supplementary Figure 3. (a, b, c) Images of figure 3 are shown without deconvolution. (d) FWHM, (e) signal intensity per area, and (f) signal intensity per puncta were quantified using the STED images without deconvolution. (a) Representative dual-color STED micrographs show clusters of Piccolo (magenta) and Bassoon (green) in single optical plane images parallel to the presynaptic membrane from NMJs of eight-month-old mice. The signal intensity profiles of these images support this distribution pattern (far right). (b) A representative dual-color STED microscopy image of Bassoon stained with two different antibodies. One antibody binds to Bassoon near the N-terminus and the other near the C-terminus. (c) The distribution pattern of Piccolo and Bassoon in NMJs of eight month old (adult) and 29 month old (aged) mice visualized using STED microscopy and shown here at low magnification. Scale bars: 100 nm for a, b, and 200 nm for c. (d) Frequency distribution graphs of Piccolo puncta size. The mean FWHM of Piccolo decreased significantly (103.8 ± 14.7 nm (adult); 99.7 ± 11.5 nm (aged), mean ± standard deviation, unpaired t-test, $p = 0.0165$, $n = 125$ (adult), 125 (aged) NMJs from five mice each). (e) Piccolo signal intensity per area was similar (adult, 13.8 ± 1.1; aged, 10.8 ± 1.0 arbitrary units, unpaired t test, $p = 0.0598$, 26 (adult), 27 (aged) NMJs from five mice each), but (f) Piccolo signal intensity per puncta decreased significantly (adult, 117.4 ± 0.7; aged, 109.3 ± 0.7 arbitrary units, unpaired t test, $p < 0.0001$, $n = 3094$ (adult), 3818 (aged) puncta from five mice each) in NMJs from aged mice compared to NMJs from adult mice.

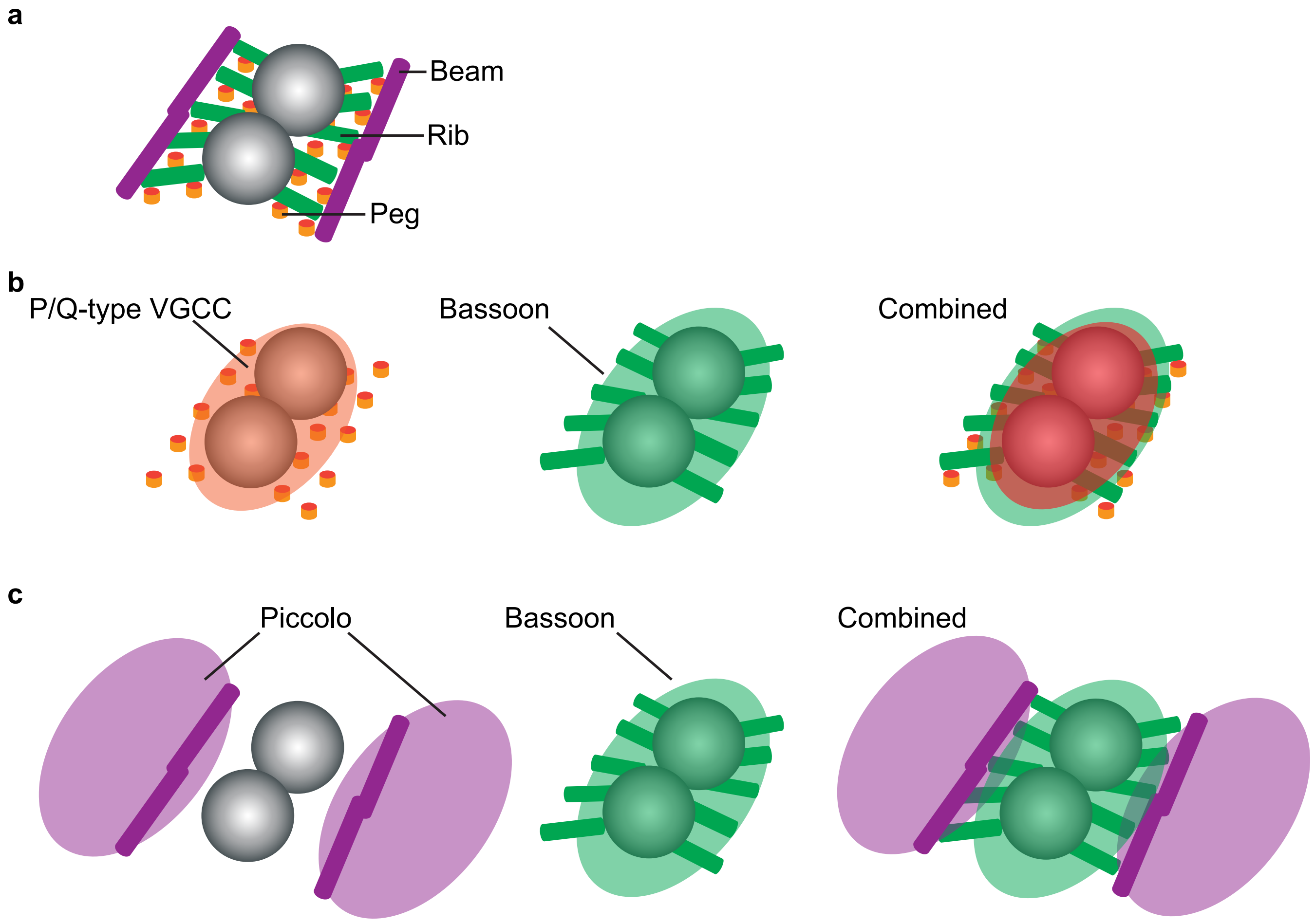

Supplementary Figure 4. A theoretical model of active zones of mouse NMJs. (a) Structure of an active zone in mouse NMJs revealed by electron microscope tomography. This schema is adapted from reference 9. Republished with permission of Dr. Uel Jackson McMahan and John Wiley and Sons Inc, from Macromolecular connections of active zone material to docked synaptic vesicles and presynaptic membrane at neuromuscular junctions of mouse, Sharuna Nagwaney, Mark Lee Harlow, Jae Hoon Jung, Joseph A. Szule, David Ress, Jing Xu, Robert M. Marshall, Uel Jackson McMahan, volume 513, issue 5, pp 467, 2009; permission conveyed through Copyright Clearance Center, Inc. (b, c) A theoretical overlay of the active zone protein distribution pattern identified in this study on the active zone structure revealed by electron tomography. The "Pegs" in Nagwaney's model are considered as transmembrane channel proteins, which may include P/Q-type VGCCs. The two ribs in Nagwaney's model were separated by about 83 nm, which is similar to the distance between two Piccolo puncta in STED images (about 100 nm).

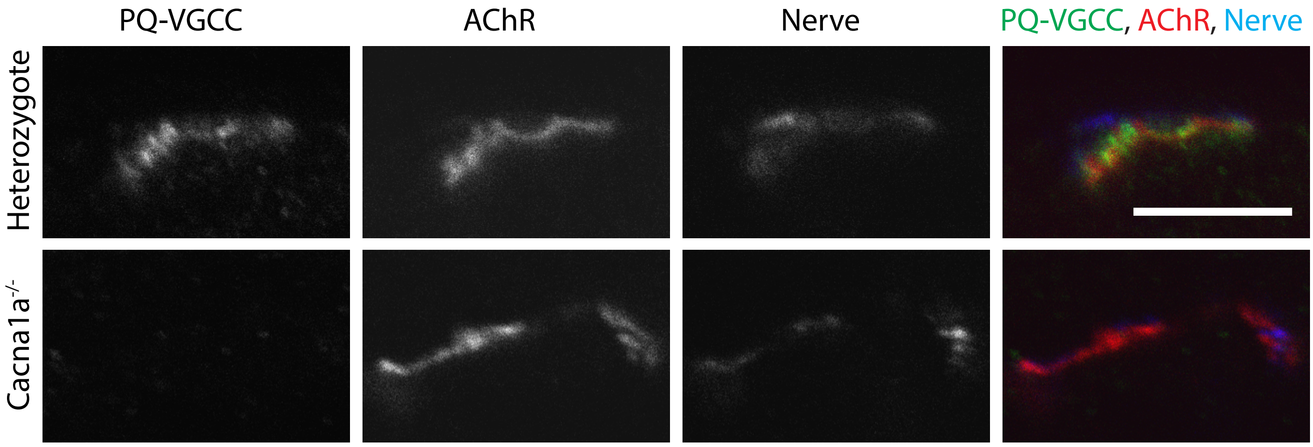

Supplementary Figure 5. P/Q-type VGCCs (PQ-VGCC, green) stained with an antibody specific for P/Q-type VGCC pore forming α subunit in NMJs of vastus lateralis muscles from postnatal day 22 mice. The sections were also stained with Alexa Fluor 594-labeled α-bungarotoxin to label acetylcholine receptors (AChR, red) and antibodies for neurofilament and SV2 to label the nerve morphology (Nerve, blue). The P/Q-type VGCC immunoreactivity is present in NMJs of heterozygote mouse for P/Q-type VGCC pore forming α subunit (Cacna1a+/-, Heterozygote), but is absent in NMJs of the littermate knockout mouse (Cacna1a-/-) demonstrating the specificity of the immunohistochemical signals. Bar: 10 μm
